# Undernutrition and associated factors among adults with mental and neurological disorders in public health hospitals, Eastern Ethiopia, 2019: a cross-sectional study

**DOI:** 10.1186/s12888-023-05117-9

**Published:** 2023-08-23

**Authors:** Samrawit Shawel, Negga Baraki, Yohanis Alemeshet, Dawit Shawel Abebe, Gudina Egata

**Affiliations:** 1 School of Public Health, College of Health and Medical Sciences, Haramaya University, Harar city, Ethiopia; 2 Department of Environmental Health Sciences, College of Health and Medical Sciences, Haramaya University, Harar city, Ethiopia; 3 Dawit Shawel Abebe, Department of Nursing and Health Promotion, Oslo Metropolitan University, St. Olavs Plass, P.O. Box 4, NO-0130 Oslo, Norway; 4 School of Public Health, College of Health Sciences, Department of Nutrition and Dietetics, Addis Ababa University, P.O.Box: 18087, Addis Ababa city, Ethiopia

**Keywords:** Body mass index, Undernutrition, Mental disorder, Ethiopia

## Abstract

**Background:**

Poor nutritional status can be consequence of impaired mental health that may lead to involuntary weight gain, weight loss, or deficiency of essential nutrients. However, little has been documented about the nutritional status of adults with mental disorders and the contributing factors in low-income countries like Ethiopia. The aim of this study was to assess the magnitude of undernutrition and associated factors among adults with mental disorders in public hospitals of Eastern Ethiopia.

**Methods:**

Institution-based, cross-sectional study was conducted among 507 adults with mental disorders from March 1, 2019 to April 1, 2019**.** Interviewer administered pretested structured questionnaire was used to collect data. Anthropometric data were collected using calibrated weighing scale and height measuring board. Descriptive statistics was computed to describe the data. Bivariable and multivariable logistic regression analyses were applied to identify factors associated with the undernutrition. Odds ratio alongside 95% confidence interval (CI) were estimated to measure the strength of the association. Level of statistical significance was declared at *p*-value less than 0.05.

**Results:**

Undernutrition affected 62.7%; 95% CI: (58.3%, 67.7%) of the patients. Undernutrition was associated with meal frequency < 3 per day (adjusted odds ratio [(AOR = 2.07, 95% CI: (1.18, 3.63)], use of multiple medication (adjusted odds ratio [(AOR = 3.02, 95% CI: (1.88, 4.84)], being non-smoker [(AOR = 0.50, 95%CI: (0.25, 0.91)], and use of prescribed diet [(AOR = 0.45, 95%CI: (0.26, 0.78)].

**Conclusions:**

The magnitude of undernutrition was high among the study participants. Multiple medication, cigarette smoking, frequency of meal and taking prescribed diet were significantly associated with undernutrition*.* Nutrition education for patients with mental disorders and their caregivers about the impact of taking multiple medication and substance use needs to be emphasized alongside nutritional screening and support to improve their nutritional status.

## Introduction

Mental health is “a state of well-being in which every individual realizes his or her own potential, can cope-up with the normal stresses of life, can work productively and fruitfully, and is able to make a contribution to her or his community” [1]. Mental disorder can be understood as” a clinically significant behavioral or psychological syndrome or pattern that occurs in a person that is associated with present distress (painful symptom) or disability (impairment in one or more important areas of functioning) or with a significantly increased risk of suffering death, pain, disability or an important loss of freedom. This syndrome or pattern must not be an expectable response to particular event” [2].

Among common mental disorders (CMDs), depressive disorders and anxiety disorders are highly prevalent and range from mild to severe, and their duration can range from months to years. Despite the fact that the prevalence of mental disorders is increasing in middle and low income countries, mental disorders don’t receive an increased attention like physical health in most part of the globe [1].

Although nutrition plays a crucial role in the onset as well as severity and duration of mental illness, the connection between nutrition and mental illness is not well known as compared with that of physical illness. Due to this reason, the field nutritional neuroscience is an emerging discipline studying the fact that nutritional factors are interconnected with human cognition, behavior, and emotions [3].

Nevertheless, available evidence reveal that there is connection between nutrition and severe mental disorders such as schizophrenia [4, 5], bipolar disorders [6] and CMDs including depression and anxiety. For instance, a study conducted in psychiatrichospitals of Japan indicated that the prevalence of underweight among schizophrenia patients was about 17.4% and 4.3% in inpatients and outpatients respectively compared to the general population (7.9%) [7].

In contrast, another study conducted in Central Taiwan Hospital, psychiatric wards among patients with schizophrenia, major depression and bipolar disorder revealed that such patients are at an increased risk of overnutrition [8]. Similarly, current data suggest that people with severe mental illness like schizophrenia have a markedly high prevalence of obesity than the general population due to their poor dietary practices [4, 5].

Evidence from systematic review suggested that dietary intake or supplementation of unsaturated fatty acids, mainly Omega-3 seems to be associated with improved bipolar disorders symptoms, along with seafood, folic acid and zinc [6]. However, patients with schizophrenia and bipolar disorders have poor dietary practices such as increased intake of sodium, cholesterol and higher saturated fats, with low fiber content, increased consumption of sugars and processed foods, and diets low in omega-3 fatty acids or D-vitamins. The unhealthy dietary practices of such patients have been suggested to result from dysregulation of the reward circuitry, mediated by increased dopamine activity in the mesolimbic pathway and brain regions responsible for the control of cognition. The development of obesity, eating disorders, food cravings and addictive behaviours observed in these patients has also been linked to the aforementioned pathway [5].

In the same line, a mini nutritional assessment (MNA) based evidence from South Africa indicated that 43.4% older South Africans with depressive symptoms were at risk of malnutrition. On the other hand, in the same study, body mass index (BMI) based assessment revealed nearly 2% of the patients were undernourished, while 30.3% and 48% of them were overweight and obese respectively [9]. Limited evidence from Ethiopia indicated that the prevalence of mild, moderate and severe undernutrition among adults with common mental disorders was 31.4%; 23.7%,4.7% and 3.0% respectively [10].

Mental disorders including depression have many consequences that may lead to poor nutritional status including involuntary weight gain, weight loss, or deficiency of essential nutrients. For instance, cardiovascular system (CVS) complications are also common due to the increased risk of being overweight/obese, physical inactivity and unhealthy lifestyles and poor nutritional status [1]. A study done in one of the Western countries showed that the rate of obesity among depressed individuals was greater than in the general population. Mental health problems are also strongly associated with risk of malnutrition in elderly individuals, and it is more prevalent among women [11]. Facility-based study indicated that 51.4% of patients with major depressive disorder had low plasma folate levels (≤ 3 ng/ml) and 50.0% of them had low plasma vitamin B12 levels (≤ 200 pg/ml) [12].

Mental disorder can be caused by several factors including genetic, psychosocial stressors, diet or nutritional, physical inactivity, drugs and environmental factors. Nutritional factors are related with the function of the brain’s neurotransmitters that are connected to a person’s cognition, behavior and emotion and may aggravate symptoms but they are not major cause. Accordingly, different studies have identified predictors of nutritional status of subjects with mental disorders which included taking multiple medication; not taking prescribed diet; living in rural area; current use of alcohol; older age; low level of education; inadequate food habit; sedentary life style and other comorbidities like high blood pressure [13–15]. Poor nutrition among these cohort of patients might be attributed to poor dietary practices, being unable to shop or prepare foods, poor access to balanced diet, unhealthy lifestyle and common antidepressants’ side effects which might lead to poor dietary intake [3].

Currently, most of the treatments of depression are psycho-pharmaceuticals but nutrition and nutrients (i.e., vitamin D, vitamin B12, vitamin C, omega 3, and probiotics) are essential to improve mental health. In contrary, saturated fats and simple sugars can be hazardous for brain health, increasing the risk for mental illnesses and also other metabolic disorders like diabetes and cardiovascular diseases [12, 14, 15]. Micronutrient intervention study indicated that the severity of depression and anxiety was significantly correlated with lifestyle factors, such as decreased or not eating of fruits and vegetables, fish, tobacco use, heavily drinking and less physical exercise [15]. The risk of depressive disorders is increased in cases of poverty, physical illness, substance use and decreased or not eating of fruits, vegetables, and fish [1, 10, 15]. A person with obesity has 55% increased risk of depression and a depressed person has 58% increased risk of obesity. The risk is increased more in obese individuals than overweight ones [11].

The efforts so far made to provide mental health care to help alleviate nutritional problems were very few especially in low and middle income countries and rarely emphasized in the national health policies [16, 17]. Moreover, compared to many nutritional research conducted among other population groups, there is limited evidence on nutritional status and associated factors among people with mental disorders in Ethiopia including the study setting. Therefore, this study aimed to determine the magnitude of undernutrition and associated factors among mentally ill adult population in public health hospitals, Dire Dawa administrative city, Eastern Ethiopia.

## Methods

### Study setting, design and period

Institutional-based cross-sectional study was conducted in public hospitals rendering mental health services for in- patients and out-patients in Dire Dawa city administration, Eastern Ethiopia. Dire Dawa city is located at about 513kms east of the capital city of Ethiopia, Addis Ababa. It has hot semi-arid climate (kola) with mean annual temperature of 25.9ºc ranging from 19.0ºc to 32.8ºc. The city is situated at an altitude of 1277 m above sea level. The population of the city is projected to be about 341,834 of which 50.2% are men and 49.8% women [18]. There are two governmental hospitals, two private hospitals, five higher clinics, twelve medium clinics (private), fifteen health centers, and thirty-four health posts in the city administration with 100% health service coverage including mental health services. The study was conducted from March 1, 2019 to April 1, 2019.

### Study participants and sample size

We included all adults with mental health problems visiting public hospitals in Dire Dawa city administration, who were admitted to the inpatient department and visiting outpatient units during the study period. However, all adult women who were pregnant or lactating for the first six months were excluded from the study since they were more likely at increased risk of undernutrition due to their increased physiological needs. Moreover, measurement of weight during pregnancy is not reliable to determine nutritional status due to weight gain during pregnancy and high caloric consumption or requirement during period of lactation. The sample size was calculated using Epi Info 7 Stat Calc computer software program for double population proportions formula, by considering known comorbidity among various factors that were significantly associated with outcome variable with the following assumptions: two-sided 95% confidence level; 80% power; proportion of outcome ( undernutrition) among exposed to be 32.3%; proportion of outcome among unexposed to be 20% [10]; an adjusted odds ratio of 1.51, and 10% for non- response yielding a sample size of 507.

### Sampling procedure

The health institutions were purposively selected since there are only two hospitals rendering mental health services in the administrative region. We included all adults seeking treatment for mental health issues at public hospitals in the study area during the study period. Accordingly, the data were collected from 289 out-patients in one government owned public hospital and 221 in-patients in another faith-based hospital offering mental health and related services in the area. Proportional allocation of the sample size was not made since the faith-based facility was very restrictive in sharing the information.

### Data collection tools, procedure and measurements

The questionnaire was initially prepared in English by reviewing relevant literature [10, 19, 20] related to the study objectives and translated to locally spoken languages, i.e., “Afaan Oromo, Amharic and Afaan Somali” for better understanding. All the three local languages were retranslated back to the original English version by their respective linguistic experts and their consistency was checked across the three languages to ascertain the content validity for the three categories of study participants to overcome an ambiguity in understanding the context based on the consensus of the experts.

Four BSC psychiatric nurses and one master of public health (MPH) holder were recruited for data collection and supervision activities respectively. Data on socio-demographic characteristics such as sex, age, marital status, religion, residence, educational status, occupational status, and living status, psychosocial, behavioral and food consumption score were collected using a pretested structured interviewer administered questionnaire. The interview was undertaken with the patient’s family or relative or legal guardian as appropriate. Weight was measured using electronic digital scale (Seca, Germany model) to the nearest 100 g and height was measured using portable height measuring board with sliding head bar to the nearest 0.1 cm following standard anthropometric measuring technique. Five contact points, head, shoulder, buttock, calves and heels, were maintained during height measurement. Body mass index (BMI) was computed as weight in Kg per height in meter square (Kg/m^2^) to determine the nutritional status of patients [21].

The outcome variable in this study is undernutrition, which refers to BMI less than 18.5 kg/m^2^ [21] and was categorized as “1” for those patients whose BMI is < 18.5 kg/m^2^ and “0” otherwise for further analysis. On the other hand, the explanatory variables included in the final analysis were sex, current living status, food intake status, frequency of meal per 24 h, taking prescribed diet, sleep problem before the diagnosis, taking multiple medication ≥ 2, past psychiatric diagnosis, known comorbidity, cigarette smoking, khat chewing, drinking wine, and drinking “areke”.

In this study, mental disorders were defined as a range of conditions that affect mood, thinking and behavior which may include depression, anxiety disorders, schizophrenia, bipolar disorders, obsessive compulsive disorder (OCD) and post-traumatic stress disorder (PTSD) [1]. The mental health diagnoses were made via clinical interviews of patients by specialists in the field who are working in the health facilities and the diagnoses were obtained from patients’ records during data collection.

Food consumption score (FCS) is a composite score which was generated based on dietary diversity, food frequency, and relative nutritional importance of 16 food groups of world food program and categorized as poor (0—28 scores), borderline ( 28.5–42) and > 42.5 as acceptable assuming that oil and sugar are eaten on a daily basis approximately 7 days per week) [19, 20, 22]. All consumption frequency of foods in the same group was summed and multiplied with value of each food group by its weight. Frequencies of food consumption equals number of days for which each food group was consumed during the past 7 days. To assess FCS, the participants were asked to recall the foods they consumed in the previous seven days before the survey. Each food item was given a score of 0 to 7 depending on the number of days it was consumed. The 16 food items were grouped into standard eight food groups with their respective weight namely Staples (Cereals and Tubers) were given weight = 2, Pulses = 3, Vegetables = 1, Fruit = 1, Meat and fish = 4, Milk = 4, Sugar = 0.5, and oil = 0.5), and the frequencies of all the food items surveyed in each food group were summed. Any summed food group frequency value over 7 was recorded as 7. For each participant, the FCS was calculated by multiplying each food group frequency by each food group weight, then summed these scores into one composite score (FCS) [22].

Multiple medication use was understood as taking greater than or equal to two anti-psychotic drugs per day. The list or number of medication was obtained from patients’ records regardless of the disaggregation of patients’ diagnosis against prescribed medication.

Substance use was defined as a pattern of repeated drug or alcohol including “areke” or tobacco use that interferes with health, work or social relationships.** “**Areke” is a traditional Ethiopian alcoholic beverage that is distilled locally. Moreover, the educational status of the study participants labeled as “no formal education” refers to those who are able to read and write on their own during the data collection.

### Data quality management

Training was given for the data collectors and supervisors for two days on the objectives of the study, interview technique, and anthropometric measurements. The standardization procedure was followed to ensure reliability and validity of anthropometric measurements by computing relative technical error of measurement (TEM) using Emergency Nutrition Assessment Standardized Monitoring and Assessment of Relief and Transitions (ENA SMART) software to compare measurements done by each data collector with selected criterion anthropometrist before deploying the data collectors to the field to minimize both random and systematic errors attributed to inaccurate anthropometric measurement. Accordingly, the relative TEM for inter-observer (validity) and intra-observer (reliability) for length/height measurement was 1.5% and 2.0%, respectively [23]. The questionnaire was pretested one week before the actual task of data collection on 5% of the estimated sample size for the study in other nearby health facility not included in the study. The whole process of data collection was supervised by supervisors in the field on daily basis for completeness of each questionnaire. Data were double entered by two data clerks and consistency was cross-checked. Multivariable analysis was done to control for all possible confounders that might mask the true association between independent and outcome variables. Moreover, the functionality of digital weight scales was checked using known weight every morning before data collection begins and before every weight measurement, the data collectors were assuring that the scale was reading exactly at zero.

### Statistical analysis

All collected data were checked for completeness and internal consistency by cross-checking and then coded and double entered onto Epi-Data version 3.1 computer software and exported to Statistical Package for Social Science (SPSS) version 22 computer software for data cleaning and analysis. Food consumption score was calculated for every individual using the food items [19]. Descriptive statistics such frequencies, mean, and proportions was used to describe all relevant data. Bivariable logistic regression analysis was carried out to see the association between each independent and the dependent variable. All variables with *p* value < 0.25 during bivariable analyses were entered into multivariable logistic regression analysis to control for all possible confounders and to identify factors associated with the outcome variable. Multicollinearity effect between explanatory variables in the final model was checked using the standard error(SE) and Pearson’s correlation test and accordingly variables with SE > 2 and correlation coefficient of ≥ 0.60 were dropped from the analysis respectively. Model fitness was checked using Hosmer and Lemeshow goodness fit test and it was found to be 0.235 indicating adequacy of the model. Odds ratio (OR) along with 95% confidence interval were estimated to measure the strength of the association. Level of statistical significance was declared at p-value lees or equal to 0.05.

## Results

### Socio-demographic characteristics of study participants

A total of 501 adults with mental disorders participated in this study yielding a response rate of 98.2%. Male study participants accounted for 62.1%. The majority (87.4%) of the respondents were younger than 45 years. The mean (± SD) age of respondents was 33.46 (± 10.8) years. Nearly less than half (42.7%) of study participants were single by marital status. About 61.1% of adults with mental disorders were Muslim religion followers. Regarding the educational status, 35.1% had no formal education. From the total study participant, 37.1% adults with mental disorders were jobless and 62.9% of them were urban residents. The majority (76%) of the study participants were from families whose average monthly income ranged from 1000 to 3000 Ethiopian birr (20–55 USD). The majority (72.1) % of the study participants were living with their families (Table 1).

**Table 1:** Socio-demographic characteristics of adults with mental health problems in public hospitals of Dire Dawa Administrative City, Eastern Ethiopia, 2019

Variables	Category	Frequency	Percent
Sex	Male	311	62.1
	Female	190	37.9
Age	18–26 years	141	28.1
	27–35 years	181	36.1
	36–45 years	116	23.2
	> 45 years	63	12.6
Current marital status	Single	214	42.7
	Married	165	32.9
	Separated	61	12.2
	Divorced	56	11.2
	Widowed	5	1.0
Religion	Muslim	306	61.1
	Orthodox	148	29.5
	Protestant	30	6.0
	Catholic	17	3.4
Living status	Alone	140	27.9
	With family	361	72.1
Educational status	No formal education **@**	176	35.1
	Primary level(1-8)	135	26.9
	Secondary (9–12)	87	17.4
	TVET^β^	51	10.2
	University level	52	10.4
Residential area	Urban	315	62.9
	Rural	186	37.1
Families average monthly income (Ethiopian birr)	< 1000	14	2.8
	1000–3000	379	75.6
	> 3000	108	21.6
Occupation	No job	186	37.1
	Private employee	60	12.0
	Government employee	56	11.2
	Farmer	95	19.0
	Merchant	40	8.0
	Daily laborer	50	10.0
	Other*	14	2.8

Other* = (student = 14) **@** = able to read and write on their own, β = Technical &Vocational Education & Training

### Dietary and substance use related characteristics

About 24.2% of adults with mental disorders reported a decrease in their food intake status after being diagnosed with mental disorders, i.e., of which 22.6% was due to loss of appetite. Most of them (74.1%) often eat meal less than three times per 24 h. A great number of them ( 89.6%) were able to feed themselves without any problem and slightly more than half of them ( 51.1%) were following their prescribed diet regimen (Table 2).

**Table 2:** Dietary habits of adults with mental health problems in public hospitals of Dire Dawa Administrative City, Eastern Ethiopia, 2019

Variable	Category	Frequency	Percentage
Food intake status	Declined	121	24.2
	No difference	380	75.8
If declined, why?	Poor appetite	113	22.6
	Lack of food	8	1.6
Frequency of feeding	< 3 times	371	74.1
	≥ 3 times	130	25.9
Feeding mode	Unable to eat without assistance	12	2.4
	Self-fed with difficulty	40	8.0
	Self-fed without any problem	449	89.6
Taking Prescribed diet	Yes	256	51.1
	No	245	48.9

**Fig. 1:**
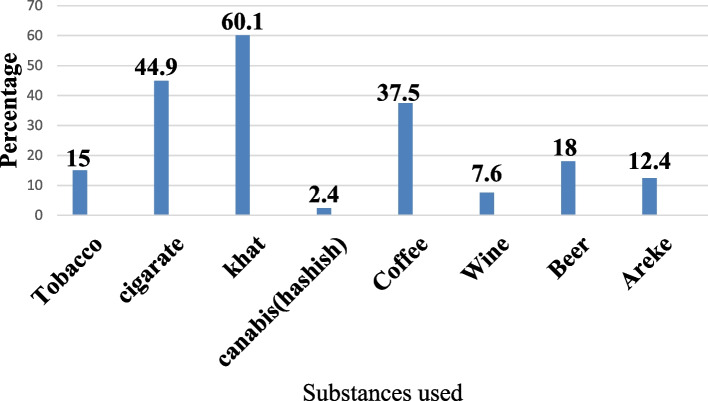
Substances and alcoholic beverages used by the study participants in the past three months, Eastern Ethiopia, 2019

More than half of the study participants (60.1%) of the study participants ever chewed Khat and almost half 44.9% of them smoked cigarette (Fig. 1).

### Psychosocial and clinical factors

More than half (62.5%) of adults with mental disorders had sleeping problem before their diagnosis, half of them were on treatment for less than three months, and 38.7% of them were getting treatment for schizophrenia. About 53.3% of the respondents were taking multiple medications (≥ 2) and 51% had been diagnosed with psychiatric disorders in the past, but the majority (76.4%) of them didn’t have any known comorbidity (Table 3).

**Table 3:** Psychosocial and clinical related characteristics of adults with mental health problems in public hospitals of Dire Dawa Administrative City, Eastern Ethiopia, 2019

Variables	Category	Number	%
Sleeping problem	Yes	313	62.5
	No	188	37.5
Length of treatment for mental health problem	< 3 months	255	50.9
	3–8 months	239	47.7
	9–27 months	7	1.4
Type of medication	Chlorpromazine	261	52.1
	Fluoxetine	69	13.8
	Sodium valconate	29	5.8
	Throdyzine	29	5.8
	Carbamazepine	63	12.6
	Phenobarbital	125	25
	Diazepam	32	6.4
	Stylizine	9	1.8
	Haloperidol	76	15.2
	Amitriypine	53	10.6
	Olanzapine	27	5.4
Taking multiple medication (≥ 2)	Yes	267	53.3
	No	234	46.7
Past psychiatric disorder	Yes	254	50.7
	No	247	49.3
Known current comorbidity	Yes	118	23.6
	No	383	76.4
Psychiatric diagnosis under treatment	Bipolar disorder	61	12.2
	Schizophrenia	194	38.7
	Brief psychosis	63	12.6
	Depression with psychotic feature	99	19.8
	Epilepsy	84	16.8

### Nutritional status of the study participants

**Fig. 2:**
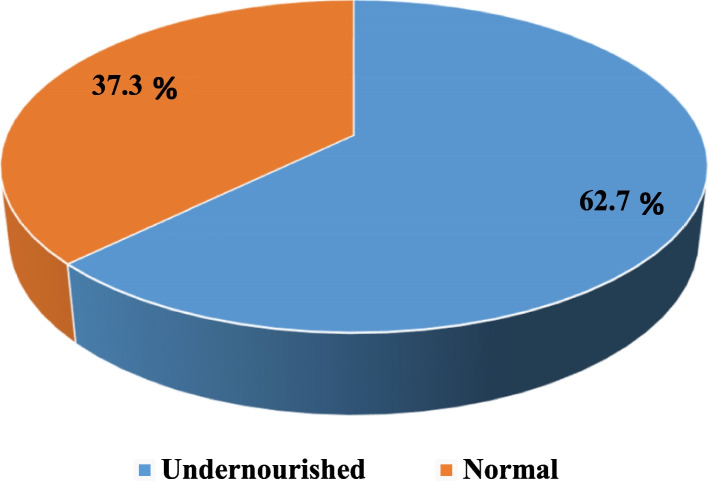
Nutritional status of adults with mental health problems in public hospitals of Dire Dawa Administrative City, Eastern Ethiopia, 2019

The mean (± SD) BMI was 18.4 (± 1.7) Kg/m^2^. From the total study participants, 62.7%, 95% CI: (58.3%, 67.7%) were underweight (Fig. 2).

### Factors associated with undernutrition among adults with mental disorders

In multivariable logistic regression analysis, frequency of meal per 24 h, taking prescribed diet, taking multiple medication and cigarette smoking were statistically significantly associated with undernutrition among adults with mental disorders. The odds of undernutrition were two times [(AOR = 2.07, 95%CI: (1.18, 3.63)] higher among adult patients whose meal frequency was < 3 times per 24 h compared with those with meal frequency of  ≥ 3 times /day. The odds of undernutrition were three times [(AOR = 3.02, 95% CI: (1.88, 4.84)] higher among adult patients using multiple medication compared with those taking a single medication. The chance of being undernourished was reduced by 53% [(AOR = 0.47, 95%CI: (0.25, 0.91)] among patients who did not smoke cigarette compared with their counterparts. Similarly, the odds of undernutrition were reduced by 55% [(AOR = 0.45, 95%CI: (0.26, 0.78)] among patients who were taking the prescribed diet compared with who did not take their prescribed diet (Table 4).

**Table 4:** Factors associated with undernutrition among adults with mental health problems in public hospitals of Dire Dawa Administrative City, Eastern Ethiopia, 2019

Variables	Category	Undernutrition		COR (95% CI)	AOR (95%CI)
		**Yes**	**No**		
Sex	Male	179(57.6%)	132(42.4%)	1	1
	Female	135(71.1%)	55(28.9%)	1.81(1.23,2.67)*	1.04(0.63,1.73)
Current living status	Alone	236(55.7%)	125(44.3%)	1	1
	With family	78(65.4%)	62 (34.6%)	0.67(0.45,0.99)*	0.80(0.50,1.30)
Food intake status	Declined	85(70.2%)	36(29.8%)	1.56(1.00,2.42)*	0.92(0.55,1.55)
	No difference	229(60.3%)	151(39.7%)	1	1
Frequency of meal per 24 h	< 3 times	265(71.4%)	106(28.6%)	4.13(2.72,6.29)*	2.07(1.18,3.63)*
	≥ 3 times	49(37.7%)	81(62.3%)	1	1
Taking prescribed diet	Yes	127(49.6%)	129(50.4%)	0.31(0.21,0.45)*	0.45(0.26,0.78)**
	No	187(76.3%)	58(23.7)	1	1
Sleep problem before the diagnosis	Yes	182(58.1%)	131(41.9%)	1	1
	No	132(70.2%)	56(29.8%)	1.70(1.16,2.50)*	1.19(0.63,2.24)
Taking multiple medication ≥ 2	Yes	190(71.2%)	77(28.8%)	2.19(1.51,3.17)*	3.02(1.88,4.84)**
	No	124(53.0%)	110(47.0%)	1	1
Past psychiatric diagnosis	Yes	146(57.5%)	108(42.5%)	1	1
	No	168(68.0%)	79(32.0%)	1.57(1.09,2.27)*	1.54(0.90,2.64)
Known comorbidity	Yes	83(70.3%)	35(29.7%)	1.56(1.00,2.44)	1.01(0.58,1.76)
	No	231(60.3%)	152(39.7%)	1	1
Cigarette smoking	Yes	202(49.8%)	74(50.2%)	1	**1**
	No	112(73.2%)	113(26.8%)	0.36(0.25,0.53)*	0.50(0.25,0.91)*
Khat chewing	Yes	175(58.1%)	126(41.9%)	0.61(0.42,0.89)*	0.66(0.36,1.23)
	No	139(69.5%)	61(30.5%)	1	1
Drinking Wine	Yes	297(44.7%)	166(55.3%)	1	1
	No	17(64.1%)	21(35.9%)	0.45(0.23,0.88)*	0.48(0.23,1.02)
Drinking Areke^@^	Yes	284(48.4%)	155 (51.9%)	1	1
	No	30(64.7%)	32(35.3%)	0.51(0.30,0.87)	0.82(0.44,1.54)

*AOR* Adjusted Odd Ratio, *CI* Confidence Interval, *COR* Crude Odd Ratio

^*^ = *p*-value < 0.05

^**^ = *p* value < 0.01@ = Locally produced Ethiopian alcoholic beverage

## Discussion

This study aimed to examine the magnitude of undernutrition and associated factors among adults with mental disorders in public hospitals in the Eastern Ethiopia. Accordingly, the magnitude of undernutrition among adults with mental disorders in the study area was nearly 63%. The predictors of undernutrition in this study included taking multiple medications, cigarette smoking, frequency of meal per 24 h and taking prescribed diet.

The observed magnitude of undernutrition in this study is relatively high as compared to prior cross-sectional studies conducted in high and middle income countries, such as Portugal 1% [13]; Japan 17.4% in inpatient and 4.3% in outpatients [7]; Turkey 6.8% in inpatient [16], and South Africa 1.8% in households at community level [9]. Moreover, another study conducted in Poland [24] indicated that 36% women and 35% of men with depressive cases and 47% women and 43% men with schizophrenia had low BMI scores, which is also greater than the result of current study. This difference could be attributed to differences in the severity of the mental disorders across studies as well as country difference in welfare and wealth status, which enables individuals to have access to basic needs and health and social care services.

The result of this study is also higher when compared with evidence from local studies in Ethiopia where undernutrition is reported to be 31.4% in Gondar [10]; 28.5% in public hospitals of North West Ethiopia [25], and 43% and 33% in Jimma among males and females respectively [26]. This discrepancy could be attributed to difference in the sample population and mode of treatment, where only outpatients were considered in latter studies. Overall, such findings imply that patients with mental disorders are subject to double burden of health problems and need special attention to promote their health.

Different factors, that have an association with undernutrition, were identified in this study. Patients who used to take multiple psychosomatic medications greater or equal to two were three times more likely to be undernourished compared with those taking single psychiatric medicine. This finding is in agreement with the study done in Poland [24] and Gondar, Ethiopia [10]. This might be attributed to the side effect of most psychosomatic drugs on the appetite of patients due to gastric upsets, decreased dietary intake and absorption leading to undernutrition.

Smoking cigarette is also associated with nutritional status of adults with mental disorders. The odds of being undernourished were reduced by half among non-smokers compared with their counterparts. This finding is comparable with a similar study conducted among elderly patients living in Tromso, Northern Norway [12]. This might be due to the fact that smoking cigarette might cause loss of appetite which in turn could lead to decreased food intake and weight loss.

Undernutrition is found to be two times more common among the study participants whose meal frequency was less than three times per 24 h compared with those whose meal frequency was ≥ 3 times per day. However, the result of other equivalent study conducted among mothers of young children with some form of distress in Democratic Republic Congo reveals that maternal mental health measures are positively associated with higher dietary diversity scores but mental health symptoms are not statistically significantly associated with nutritional status of the mothers [27]. We recommend that this discrepancy about the impact of dietary diversity needs to be further investigated.

Moreover, the odds of undernutrition were reduced by 55% among patients who correctly consumed diet prescribed by their psychiatrists compared with their counterparts. This finding is consistent with the study conducted in Gondar, Ethiopia [25]. The possible explanation could be that professionally prescribed diet may improve the nutritional status of patients. It takes into account diet that is rich in all important nutrients to satisfy the body’s required calorie needs.

The overall results of this study imply that the nutritional status of adults with mental disorders need a great attention since these group of people are very much left out of the national nutrition program especially in low-income countries like Ethiopia. The study showed the majority of these adult patients are affected by undernutrition. Nutrition intervention programs should thus target this cohort of patients to improve their nutritional status and to have a good outcome in the treatment of mental disorders.

This study may have the following limitations: Firstly, an anthropometric measurement error could misclassify the nutritional status of the study participants. However, data collectors were well trained, standardization of anthropometric measurements was done and the measuring instrument was calibrated to minimize the technical error of measurement. Secondly, since the respondents to our research questions were patients’ family/relative or legal guardians for some cases, there could be a possibility that some of the responses might suffer from recall bias and reporting bias affecting the point estimate. However, interviewers allowed sufficient time for the parents/relatives/legal guardians to adequately recall long-term memory. Lastly, the food consumption score was calculated for every respondent but all of the respondents score was < 28 which implies poor score. This was because the majority of the data were collected from inpatients living in the treatment institution eating similar type of diet with the same frequency and the time of data collection was fasting period for religious purpose. Most of the Christian patients did not consume animal source foods.

In conclusion, the result of this study indicates that magnitude of undernutrition among adults with mental disorders in the study setting was found to be high. Taking multiple medications, cigarette smoking, frequency of meal per 24 h and taking prescribed diet are significant predictors of undernutrition. Educating patients and their family/relatives/legal guardians about the impact of taking multiple medication and substance use needs to be emphasized by all relevant actors alongside nutritional support and screening at facility level to improve the nutritional status of mentally ill individuals. We would also like to recommend further large scale study for a better understanding of the nutritional status of mentally ill individuals to design appropriate interventions at all levels.

## Data Availability

The datasets generated and/or analysed during the current study are not publicly available due to privacy but are available from the corresponding author on reasonable request.

## Abbreviations

AOR: Adjusted odds ratio
BMI: Body mass index
CI: Confidence interval
COR: Crude odds ratio
CVS: Cardiovascular system
ENA SMART: Emergency nutrition assessment standardized monitoring and assessment of relief and transitions
Kg/M^2^: Kilogram per meter square
mph: Master of public health
PTSD: Post-traumatic stress disorder
SD: Standard deviation
SPSS: Statistical package for social science
TEM: Technical error of measurement
WHO: World health organization
